# Structure-mechanics statistical learning uncovers mechanical relay in proteins†

**DOI:** 10.1039/d1sc06184d

**Published:** 2022-01-19

**Authors:** Nixon Raj, Timothy H. Click, Haw Yang, Jhih-Wei Chu

**Affiliations:** Institute of Bioinformatics and Systems Biology, National Yang Ming Chiao Tung University Hsinchu 30010 Taiwan Republic of China; Department of Chemistry, Princeton University Princeton NJ 08544 USA; Institute of Bioinformatics and Systems Biology, Department of Biological Science and Technology, Institute of Molecular Medicine and Bioengineering, Center for Intelligent Drug Systems and Smart Bio-devices (IDS^2^B), National Yang Ming Chiao Tung University Hsinchu 30010 Taiwan Republic of China jwchu@nctu.edu.tw

## Abstract

A protein's adaptive response to its substrates is one of the key questions driving molecular physics and physical chemistry. This work employs the recently developed structure-mechanics statistical learning method to establish a mechanical perspective. Specifically, by mapping all-atom molecular dynamics simulations onto the spring parameters of a backbone-side-chain elastic network model, the chemical moiety specific force constants (or mechanical rigidity) are used to assemble the rigidity graph, which is the matrix of inter-residue coupling strength. Using the S1A protease and the PDZ3 signaling domain as examples, chains of spatially contiguous residues are found to exhibit prominent changes in their mechanical rigidity upon substrate binding or dissociation. Such a mechanical-relay picture thus provides a mechanistic underpinning for conformational changes, long-range communication, and inter-domain allostery in both proteins, where the responsive mechanical hotspots are mostly residues having important biological functions or significant mutation sensitivity.

## Abstract 2

Protein residues exhibit specific routes of mechanical relay as the adaptive responses to substrate binding or dissociation. On such physically contiguous connections, residues experience prominent changes in their coupling strengths.

## Graphical abstract

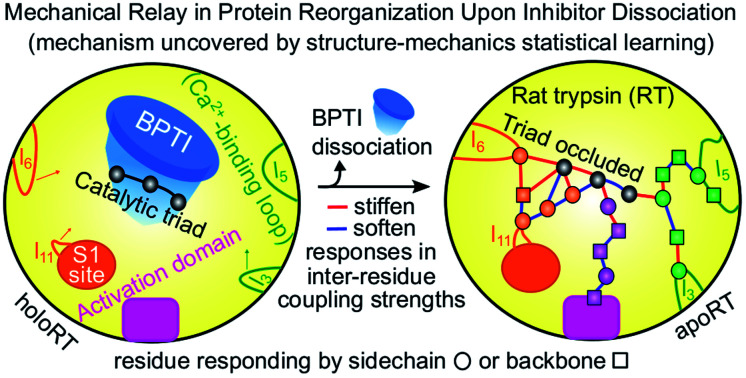

## 1 Introduction

Upon binding a specific molecule such as an inhibitor or a substrate, the interaction network in a protein is expected to self-adjust for the biological function.^1–3^ Even without a conformational change, reorganization can proceed as variations in its dynamical fluctuations.^4–6^ Although adaptive responses to molecular binding have long been anticipated from structural, biochemical, and spectroscopic measurements,^7–9^ this functionally very important property is mostly understood phenomenologically. Quantitative metrics based on physical interactions are lacking due to the difficulties of observing molecular scale processes in a complex system. For example, the respective roles of backbone and side chains in open/close structural transitions that gate an enzyme active site are yet to be resolved.^10–12^

**Fig. 1:**
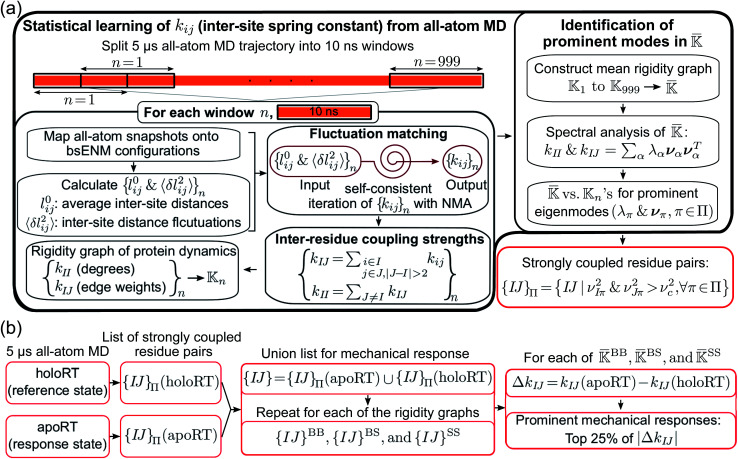
Flowcharts for statistical learning of protein reorganization upon substrate binding or dissociation. (a) Computation of inter-site spring constants (*k*_*ij*_'s) in the backbone-side-chain elastic network model (bsENM) from all-atom MD, construction of rigidity graphs with *k*_*ij*_'s, identification of their statistically prominent eigenmodes (Π = {π}), and generation for the list of strongly coupled residue pairs, {*IJ*}_Π_, from π ∈ Π (red box). (b) Construction of a union list {*IJ*}from the {*IJ*}_Π_ at different substrate binding states. Given the union lists of 
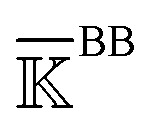
, 
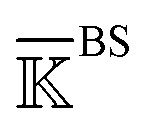
, and 
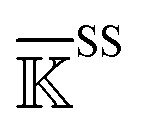
, protein reorganization is the differences in their matrix components. The top 25 percentile of |Δ*k*_*IJ*_| in a union list are the prominent mechanical responses of the rigidity graph upon molecular binding. BPTI unbinding of RT is used here for illustration.

To understand protein reorganization in terms of molecular interactions, a mechanical-coupling dynamics perspective is taken here. According to the equipartition principle in statistical mechanics—the extent of fluctuation inversely reflects the interaction strength—a structure-mechanics statistical learning scheme can be developed to compute the parameters of a coarse-grained (CG) backbone and side-chain elastic network model (bsENM) from the configurations in an all-atom molecular dynamics (MD) trajectory.^13,14^ Using the calculated elastic constants as edge weights between residue nodes gives the rigidity graph of protein dynamics, Fig. 1(a). The mechanical couplings of both backbone and side chains have been shown to exhibit scale-free network properties,^14^ indicating that only a fraction of structural contacts exhibit strong interactions. Over the μs time-scale MD conducted in explicit solvent, the statistically prominent eigenmodes of a protein rigidity graph reveal the specific molecular interaction patterns that persist through stochastic noises.

The unique network features of inter-residue interaction strengths uncovered at a particular state of substrate binding^14^ provide a remarkable opportunity for learning the mechanism of protein reorganization by avoiding the difficulties seen in covariance of structural fluctuations^15–17^ or sequence changes,^18–20^ in which the signals oftentimes do not come from physical interactions. For example, the apparent correlation between distal residues in soft vibrational modes is largely influenced by the topological shape of the native fold.^21–23^ Inferring the coupling strengths from the correlated fluctuation of each residue pair^24^ is also complicated by its intricate connections in the structure. Collective consideration of the other degrees of freedom, such as by the self-consistent iterations in structure-mechanics statistical learning,^14^ is necessary to quantify the sparse mechanical coupling network. In this case, the prominent eigenmodes of rigidity graphs correspond to persistent restraints coming from hydrogen bonding, electrostatic couplings, and hydrophobic contacts.^14^ Therefore, comparing the rigidity graphs from the protein dynamics of different molecular binding states is an appealing strategy to study protein reorganization.

This approach is taken here to study the reorganization of a serine protease family member rat trypsin (RT) upon unbinding the BPTI inhibitor and of the third PDZ signaling domain (PDZ3) after binding the CRIPT peptide substrate. In RT, substrate variation or mutation at sites away from the catalytic triad still impact its activity; yet, how such a long-range communication is achieved is not clear.^25–29^ Whereas for PDZ3, the linkage between its intra-domain^5,6^ and inter-domain allostery^30^ has not been established.

## 2 Materials and methods

The computational framework integrating explicit-solvent all-atom MD simulations, structure-mechanics statistical learning, and graph theory is summarized in Fig. 1. After the calculation of rigidity graph from protein dynamics and identification of the set of statistically prominent eigenmodes, Π, the list of strongly coupled residues pairs, {*IJ*}_Π_, is determined as shown in the lower-right corner of Fig. 1(a). Next, the scheme devised here for comparing the protein mechanical coupling networks at different substrate binding states is summarized in Fig. 1(b). The different components of this approach are discussed in more detail in the following.

### 2.1 All-atom MD at different substrate binding states

The X-ray structure of RT bound with BPTI (holoRT, PDB ID: 3TGI)^31^ is used to construct the all-atom model of apoRT based on the procedure^14^ of model development for holoRT. For PDZ3, the 1BE9 X-ray structure^32^ is used to construct the model of holoPDZ3 following the steps of setting up apoPDZ3 (ref. 14) with 1BEF. All systems are solvated in orthorhombic dodecahedron TIP3P water boxes and neutralized with NaCl ions at 0.15 M. The CHARMM36 all-atom force field^33^ is used to compute the potential energy and the GROMACS software^34^ is used for MD runs. After equilibration, the production run is at 300 K and 1 atm for 5 μs for all simulations, during which a snapshot is saved every 1 ps for analysis. In probing variation in the protein mechanical coupling network, holoRT and apoPDZ3 are taken as the reference states and their trajectories were collected as reported in ref. 14. Here, the same simulation protocol is used for the all-atom MD simulations of the response states, apoRT and holoPDZ3.

### 2.2 Calculation of rigidity graphs from protein dynamics

The 5 μs all-atom MD production run is obtained for both the apo and holo states of each protein system. Each trajectory is split into overlapping 10 ns windows as shown in Fig. 1(a), and the atomistic configurations in each are used to compute the spring parameters (equilibrium length *l*_*ij*_^0^ and spring constant *k*_*ij*_ between each site pair in the potential energy function) of the coarse-grained (CG) backbone-side chain elastic network model (bsENM) as defined in ref. 14. This split of a long trajectory into window segments is to overcome the harmonic approximation of bsENM. The structure-mechanics statistical learning method involves self-consistent iterations with normal mode analysis (NMA) to compute the length fluctuation of each spring, 〈*δl*_*ij*_^2^〉_NMA_, and match the corresponding fluctuation observed in all-atom MD simulation, 〈*δl*_*ij*_^2^〉_AA_. In each trajectory window, the equilibrium length *l*_*ij*_^0^ is the averaged distance between CG sites *i* and *j* in the all-atom MD segment. For an isolated spring, the variance of length fluctuation at thermal equilibrium is inversely proportional to the magnitude of spring constant according to the equipartition theorem. Given the system of coupled springs in bsENM, the elastic parameters are thus updated as 
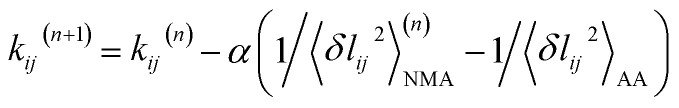
 with (*n*) the iteration number and *α* the learning rate.^35^ The other components of the structure-mechanics statistical learning, including the determination of cutoff length for including a spring in bsENM and the quantitative calibration, are reported in ref. 14.

The rigidity graphs of protein dynamics are constructed from the statistically learned inter-site force constants, *k*_*ij*_'s. Residues indexed by *I* and *J* are nodes and the inter-residue coupling strengths *k*_*IJ*_'s are edge weights. The inter-residue coupling strength is calculated as 
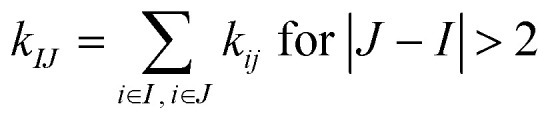
 and zero otherwise to focus on the strengths of non-covalent interactions and the springs of disulfide bonds are also excluded. With the degree as 
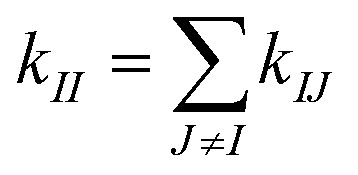
, a rigidity graph 
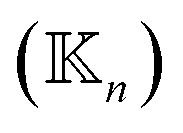
is constructed for each *n* of the trajectory windows. Since the bsENM is composed of backbone and side-chain sites, the rigidity graph can be categorized as backbone–backbone 
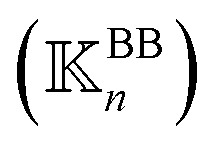
, backbone-side chain 
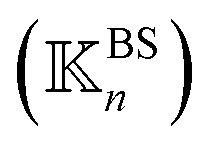
and side chain-side chain 
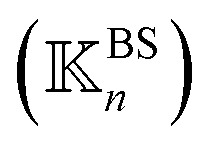
.

### 2.3 Statistical analysis of rigidity graphs

For statistical analysis of the fluctuating rigidity graphs along the all-atom MD trajectory, we consider the mean rigidity graph by averaging over those of each trajectory window, *i.e.*, 
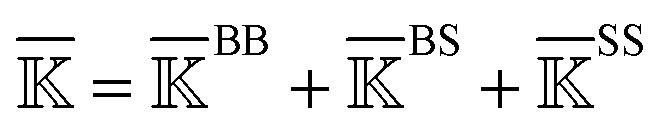
. For each type (BB, BS, or SS) of the mean rigidity graph 
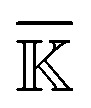
, spectral decomposition is conducted and 
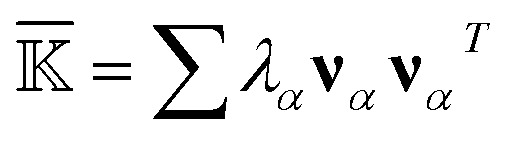
. Here, the superscript is dropped for simplicity, and this analysis is conducted for each of 
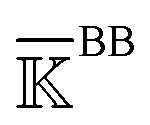
, 
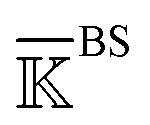
, and 
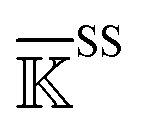
. In each case, the mean modes have the eigenvectors (***ν***_*α*_’s) and eigenvalues (*λ*_*α*_’s). Each mean mode *α* is then compared with all the modes of every window to compute its mean-mode content in the window as *r*_*nα*_ = max_*β*_|***ν***_*nβ*_·***ν***_*α*_|. Averaging the mean-mode contents of all gives the averaged mean-mode content 〈*r*_*α*_〉.

The prominent modes of a particular rigidity graph, are then identified by two criteria.^14^ The first is to find the mechanically strong modes with a high eigenvalue (statistical outliers of the *λ*_*α*_ distribution). The second is to identify the highly persistent eigenvectors by fitting the distribution of 〈*r*_*α*_〉 and getting the values greater than a cumulative density function (CDF) cutoff. The modes that satisfies both these conditions are the prominent modes of ultra-high mechanical coupling strengths that persist through thermal noise in the all-atom MD trajectory. From each member in the set of prominent modes Π, the strongly coupled residue pairs are identified as both residues taking a weight higher than a cutoff in the eigenvector, 
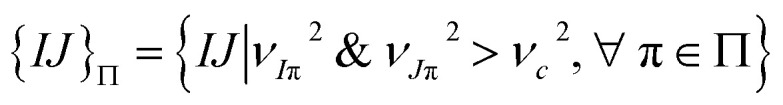
 and putting all such residues together forms the list of strongly coupled residues pair in the rigidity graph, *i.e.*, {*IJ*}_Π_ in Fig. 1(a). For consistent comparison of rigidity graphs, the numerical details reported in ref. 14 (*ν*_*c*_^2^ = 0.1) are employed here for the alternative substrate binding state.

### 2.4 Identification of mechanically responsive residues upon substrate binding

Since the protein mechanical coupling network depends on the state of inhibitor or substrate binding, the list of strongly coupled residues as in the prominent modes of rigidity graph, {*IJ*}_Π_, would also vary. As shown in Fig. 1(b), the identification of mechanically responsive residues upon substrate binding starts by finding the union list {*IJ*} from the prominent rigidity graph modes at the reference state and the response state. For BPTI unbinding of RT, the reference state is holoRT and the response state is apoRT. From the union list of strongly coupled residues {*IJ*}, variation in the mechanical coupling network is measured as Δ*k*_*IJ*_ and the prominent mechanical responses are residue pairs within top 25% of the |Δ*k*_*IJ*_| values, Fig. 1(b).

### 2.5 Multiple sequence alignment for PDZ3

The third PDZ domain of PSD-95 (PDZ3) has a CT-extension α-helix, α_3_, packed against the β-sandwich which acts as a part of the linker between the third PDZ domain and the SH3 domain.^36^ To identify the PDZ3 sequences possessing the α_3_ extension, the subrange sequence from H372 to A402 of 1BE9 is used to BLAST against the non-redundant database excluding the models and uncultured/environmental samples. A seed length of 3 is used to increase the specificity against the sequence database which resulted in 1457 sequences. The hypothetical, clone variants and other redundant sequences are the removed, resulting in the final 45 sequences with diverge taxonomic lineage. The multiple sequence alignment of these strings is conducted by T-Coffee.^37^

## 3 Results and discussion

Beginning with the case BPTI unbinding in RT, reorganization in the mechanical coupling network is captured based on the rigidity graphs of different chemical components (BB, BS, and SS) that all have the same dimension as the total residue number. For a particular rigidity graph, the prominent mechanical responses—top 25 percentile of the |Δ*k*_*IJ*_| values in the union list {*IJ*} (holoRT and apoRT) of strongly coupled residues pairs—are softening (Δ*k*_*IJ*_ < 0) or stiffening (Δ*k*_*IJ*_ > 0), and the rest are neutral. Putting together the responses of Δ*k*^BB^_*IJ*_, Δ*k*^BS^_*IJ*_, and Δ*k*^SS^_*IJ*_ uncovers the specific routes of mechanical relay. Whether such intriguing patterns would appear in a different functionality of signaling is then analyzed by comparing the rigidity graphs learned from the protein dynamics of holoPDZ3 and apoPDZ3.

### 3.1 Occlusion of RT active site upon BPTI unbinding

**Fig. 2:**
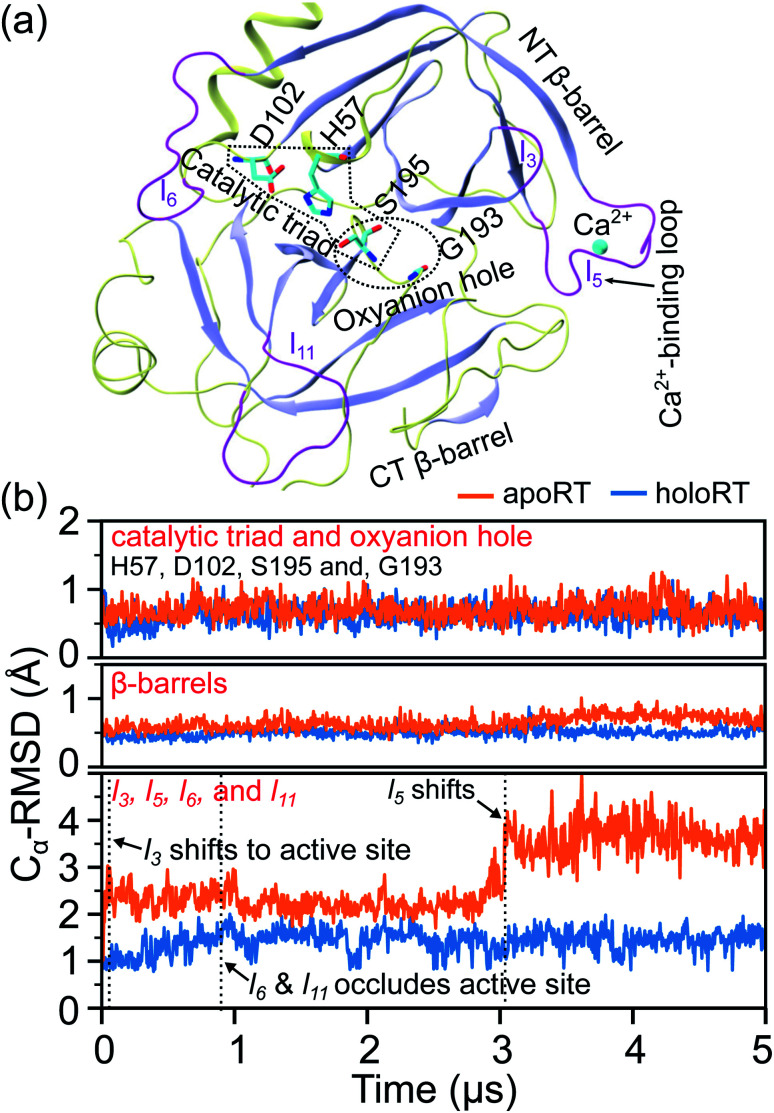
The C_α_ RMSD (root-of-mean-squared-deviation) of RT from the coordinates in the reference X-ray structure in holoRT and in apoRT simulations. (a) A ribbon representation of the RT structure with the β-barrels colored in iceblue and the NT- and CT-barrels labelled. The catalytic triad and oxyanion hole at the β-barrel interface are shown in licorice. The Ca^2+^ ion is shown in a ball representation. (b) The C_α_ RMSD of catalytic triad and oxyanion hole residues (top), the β-barrel residues (middle), and the surface loops exhibiting active site occlusion upon BPTI unbinding and the Ca^2+^-binding loop (bottom).

Over the 5 μs trajectories of holoRT and apoRT, the active site residues involving the triad and the oxyanion hole (Fig. 2(a)) remain close to the conformation in the reference X-ray structure as their C_α_ RMSD (root-of-mean-squared-deviation) values are small (∼1 Å), Fig. 2(b). Similarly, the inhibitor unbinding does not affect the β-barrel structures in RT and they have very small RMSD values throughout the production runs. The conformational responses, though, mostly occur at surface loops and their C_α_ RMSD values are significantly higher, Fig. 2(b). As the space occupied by BPTI becomes available in apoRT, specific residues in these regions shift to interact with active site residues. Furthermore, even the *l*_5_ loop, *i.e.*, the Ca^2+^-binding loop, distal to the active site exhibits significant conformational changes in apoRT. To understand such behaviors in terms of physical interactions, the protein reorganization is analyzed based on the prominent changes in the mechanical coupling network.

### 3.2 RT reorganization exhibits routes of mechanical relay

**Fig. 3:**
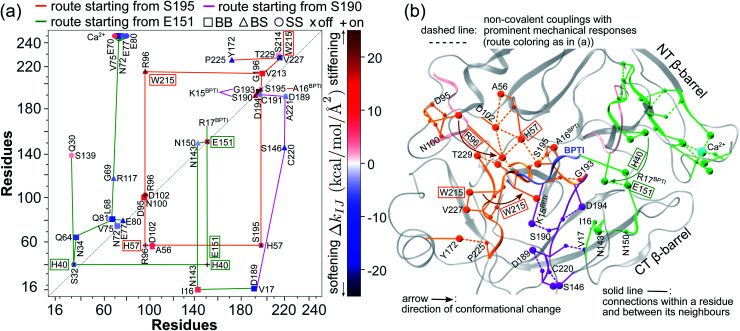
Reorganization of the mechanical coupling network in RT upon BPTI unbinding. (a) The prominent mechanical responses of inter-residue couplings—top 25 percentile of the |Δ*k*_*IJ*_| values in the union list of the strongly coupled pairs in each rigidity graph, 
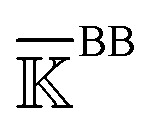
 (square), 
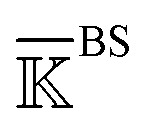
 (triangle), and 
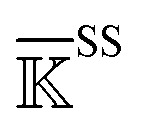
 (circle). The levels of softening (Δ*k*_*IJ*_ < 0, blue) and stiffening (Δ*k*_*IJ*_ > 0, red) responses are represented by the color bar. The prominent couplings with BPTI in holoRT are labelled on the diagonal. The key RT residue interacting with BPTI that starts a specific route of mechanical relay is used to annotate the chains of interaction network reorganization. If *k*_*IJ*_ = 0 in the response state, the pair is labelled “off ” with ×. If *k*_*IJ*_ = 0 in the reference state, the pair is labelled “on” with +. (b) The residues of mechanical relay systems in (a) on the RT structure.

The prominent mechanical responses in the RT rigidity graphs are shown in Fig. 3(a) with a color bar to quantitatively indicate the stiffening/softening levels of inter-residue couplings. Marking the significant Δ*k*_*IJ*_ values on the corresponding residue indices reveals physical contiguity in the prominent mechanical responses. In Fig. 3(a), a line is put between two marks if a common residue is shared or if they have residues of nearby sequences (mostly ≤ 2, no more than 4 residues). Consecutive connection of such lines leads to the routes in Fig. 3(a) that have nearly vertical and/or horizontal sections. On the RT structure, residues participating in the prominent mechanical responses are shown in Fig. 3(b).

**Fig. 4:**
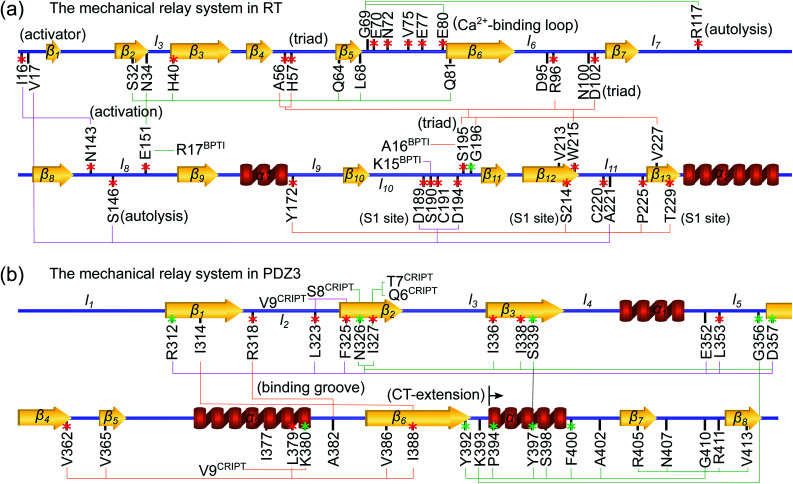
Functional and evolutionary significance of protein intramolecular mechanical relays. Mechanically responsive residues in molecule-binding induced reorganization are mostly functional sites identified in experiments (red star), or have high sequence conservation (green star). The mechanical relay systems identified in Fig. 3 for RT and in Fig. 6 for PDZ3 are listed here on the primary sequence with secondary structures annotated. (a) In RT, H57, D102, S195, and G193 are the catalytic triad and oxyanion hole, respectively;^29^ nearby D189 and S214 are the S1 site for specificity control in substrate binding;^29^ R117 and S146 are protease autolysis sites;^38–40^ H40,^41^ E70,^42^ and W215 (ref. 43–45) at regulatory loop edges exhibit long-range effects on activities; R96 in a surface loop conducts active site occlusion in a homolog;^46,47^ with I16 and D194 activators forming a salt bridge, the activation domain involves N143 in *l*_8_, D189-C191 in *l*_10_, and C220 in *l*_11_;^41^ and functional mutation sensitivity has been demonstrated for the other labelled residues.^48–50^ (b) In PDZ3, the identified functional sites showing mutation sensitivity are mostly in the β-sandwish core.^51,52^ Including CT-extension in MSA as motivated by their inter-domain couplings in the rigidity graphs reveals the highly conserved residues shown here.

Here, the reference state is holoRT and apoRT is the response state, Fig. 1(b). From the all-atom MD trajectory of holoRT, the strong couplings with BPTI were identified to be 
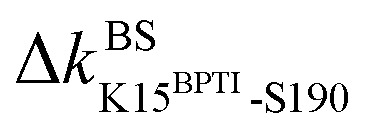
, 
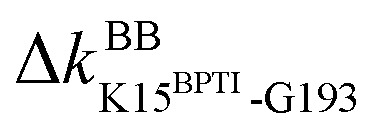
, 
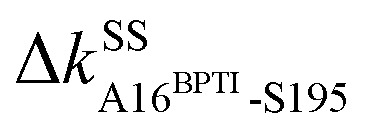
, and 
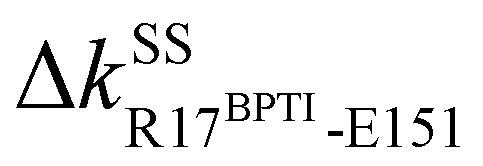
.^14^ Since they become absent in apoRT, the off mark “×” is labeled on the diagonal of Fig. 3(a) at the corresponding residues of RT. In capturing the prominent mechanical responses in RT upon the inhibitor unbinding (Fig. 1(b)), the significant Δ*k*_*IJ*_ changes are obtained by comparing the residue pairs in the protease. In Fig. 3(a), the off mark is also labelled over the intra-RT couplings that are lost upon BPTI unbinding. On the other hand, the on mark “+” indicates the strong interactions formed in apoRT but not in holoRT. In RT, S195, D102, and H57 are the catalytic triad, and G193 is the oxyanion hole. The secondary structures and functional sites of RT are annotated in Fig. 4(a).

Considering the reorganization branching from S195 with 
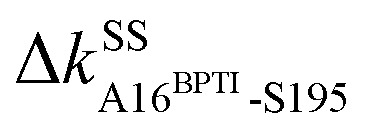
 off in apoRT, the neighboring Δ*k*^BB^_G196-V213 _stiffens, Fig. 3(a). W215 nearby V213 at the C-terminal end of β_12_ then moves toward the active site (Fig. 3(b)) and detaches V227 at S1 site, *i.e.*, Δ*k*^SS^_W215-V227 _off. This positional shift of the W215 side chain at the end of β_12_ and beginning of *l*_11_ that softens the nearby couplings at the S1 site is coupled with R96 in *l*_6_, which also migrates to the space originally occupied by BPTI and turns on Δ*k*^SS^_R96-W215_, Fig. 3(a). With R96 coupling two triad residues (Δ*k*^SS^_R96-D102_ and Δ*k*^SS^_R96-H57_) through this conformational change in apoRT, Δ*k*^SS^_H57-S195 _is switched off in the response state. Therefore, losing the interactions with BPTI leads to a chain of adjustment in the interaction network that eventually occludes the catalytic triad in apoRT by W215 and R96. This route of coupling strength variation over contiguous residues—a system of mechanical relay—is colored orange in Fig. 3 and 4. In this case, W215, R96, and H57 act as on-off switches by interacting with alternative partners at different states of inhibitor binding. In the S1A protease family, allosteric active site occlusion has been observed for the W215 loop in thrombin^43–45^ while for the R96 containing loop in coagulation factor IXa and in trypsin-like hepatocyte growth factor activator.^46,47^ The previously unknown mechanism as identified here for RT is the route of mechanical relay over spatially contiguous residues.

Another scenario is E151 that strongly interacts with R17^BPTI^ in holoRT. As 
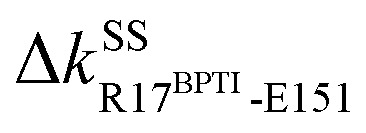
 becomes off in apoRT, the imidazole side chain of H40 at the start of β_3_ shifts to interact with E151 and Δ*k*^SS^_H40-E151 _is turned on. This conformational change also shuts off Δ*k*^SS^_S32-H40 _in apoRT, and the E151 and H40 mechanical switches start a chain of softening responses that reach the Ca^2+^-binding loop around E70. This distal loop has been shown to relate to the long-term stability of family members and exhibit stimulatory effects on enzyme activities.^42^ For example, in urokinase-type plasminogen activator, communication between H40 and E70 loops has been implied from the inhibitor binding and enzyme activity experiments.^41^ Our finding in RT uncovers the underlying mechanism as the domino-like variation in mechanical couplings. This route seeding from E151 also involves a reported autolysis site R117 (ref. 38–40) and is colored green in Fig. 3 and 4(a).

### 3.3 Hotspots in mechanical relay capture functional sites

Functional regulation in many S1A proteases has been found to proceed by movements of surface loops and the participating segments are a key signature for member classification.^42^ The previously unknown behaviors of RT are uncovered here to have a unique profile, including *l*_6_ of R96, *l*_11_ starting at W215, *l*_3_ ends at H40, and the Ca^2+^-binding loop, Fig. 3. Along a route of mechanical relay, an important notice is that having a conformational change is not necessary. For example, as 
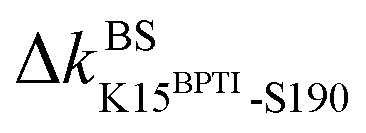
 and 
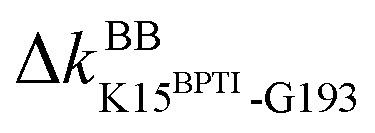
 become off in apoRT, the pathway colored purple in Fig. 3 and 4 involves soften responses without having any on-off switch. S146, another reported autolysis site in thrombin and bovine trypsin,^38–40^ is on this route.

An intriguing finding in the case of RT reorganization upon BPTI unbinding is that the mechanically responsive residues during the different protein dynamics are mostly the functional sites identified experimentally, Fig. 4(a). For example, the salt bridge between I16 and D194 retains similar strengths in both holoRT and apoRT trajectories, but the two biologically important residues exhibit significant variation in their strengths of the couplings with other partners as seen on the purple route in Fig. 3 and 4(a). Occurring through specific chains of physically contiguous amino acids, the significant coupling strength variation in the interaction network reorganization of all-atom MD simulations—the hotspots of mechanical relay—is thus a useful metric for the functional importance of residues.

### 3.4 PDZ3 exhibits mechanical relay upon binding CRIPT

**Fig. 5:**
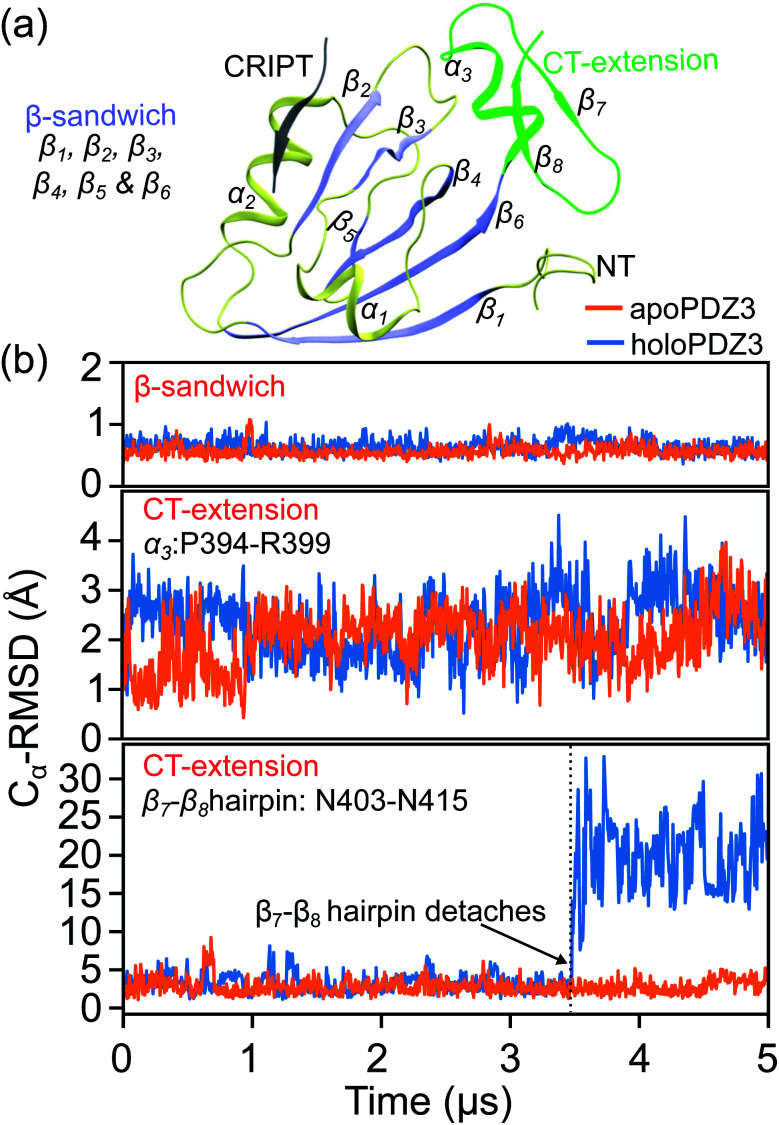
The C_α_ RMSD (root-of-mean-squared-deviation) of PDZ3 from the coordinates in the reference X-ray structure in holoPDZ3 and apoPDZ3 simulations. (a) A ribbon representation of the PDZ3 structure with the β-sandwich colored in iceblue. The CT extension is colored green and the CRIPT peptide is colored gray. (b) The C_α_ RMSD of β-sandwich (top), the α_3_-helix in CT-extension (middle), and β_7_–β_8_ hairpin in CT-extension (bottom).

To study whether similar behaviors could be observed in an alternative functionality of signaling rather than in an enzyme, the self-adjustment in PDZ3 upon binding the CRIPT peptide is analyzed by comparing the rigidity graphs holoPDZ3 (response state) and apoPDZ3 (reference state) following the schemes outlined in Fig. 1. With the binding groove in between β_2_, *l*_2_, and α_2_, Fig. 4(b) and 5(a), the β-sandwich core has been shown to conduct intra-domain allostery without structural changes.^5,6^ Substrate binding in PDZ3 also impacts C-terminal (CT)-extension conformation as illustrated by NMR and SAXS measurements,^53^*i.e.*, exhibiting inter-domain allostery.

Over the 5 μs production runs of the all-atom MD simulation of apoPDZ3 and holoPDZ3 in explicit solvent, the β-sandwich stays close to the reference X-ray structures^32^ as illustrated by the small C_α_ RMSD values in Fig. 5(b). The α_3_ helix in CT-extension is more flexible, but remains attached to the β-sandwich in both apoPDZ3 and holoPDZ3. On the other hand, the β_7_–β_8_ hairpin in CT-extension remains attached to the β-sandwich throughout the apoPDZ3 reference state, but in holoPDZ3, it detaches and loosens (Fig. S1†), showing pronounced RMSD values in Fig. 5(b). This is a first atomic-scale *in silico* observation that the substrate binding in PDZ3 affects the conformation of distal CT-extension as speculated in NMR and SAXS studies.

**Fig. 6:**
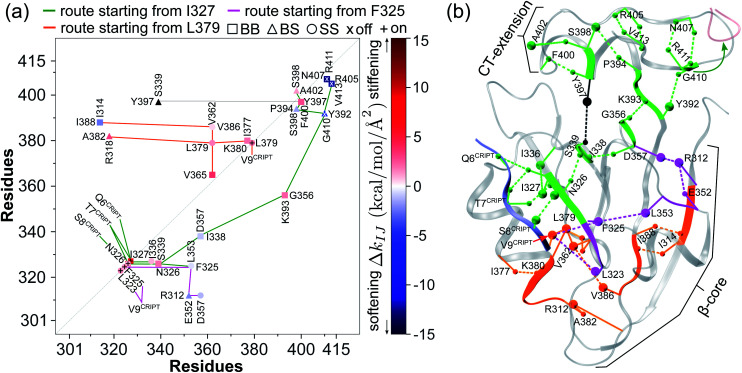
Reorganization of the mechanical coupling network in PDZ3 upon binding the CRIPT peptide. (a) The prominent mechanical responses of inter-residue couplings—top 25 percentile of the |Δ*k*_*IJ*_| values in the union list of the strongly coupled pairs in each rigidity graph, 
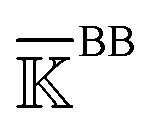
 (square), 
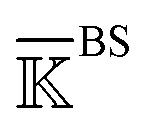
 (triangle), and 
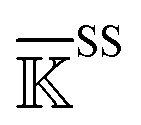
 (circle). The levels of softening (Δ*k*_*IJ*_ < 0, blue) and stiffening (Δ*k*_*IJ*_ > 0, red) responses are represented by the color bar. The prominent couplings with CRIPT in holoPDZ3 are labelled on the diagonal. The key PDZ3 residue interacting with CRIPT that starts a specific route of mechanical relay is used to annotate the chains of interaction network reorganization. If *k*_*IJ*_ = 0 in the response state, the pair is labelled off with ×. If *k*_*IJ*_ = 0 in the reference state, the pair is labelled on with +. (b) The residues of mechanical relay systems in (a) on the PDZ3 structure. Definitions of sold/dash lines and arrows are as in Fig. 3(a). The S339-Y397 coupling prominent in both apoPDZ3 and holoPDZ3 is colored black.

The underlying mechanism of this inter-domain, long-range effect of CRIPT binding is unraveled here by comparing the rigidity graphs of different protein dynamics, *i.e.*, holoPDZ3 *versus* apoPDZ3. In the former, T7^CRIPT^ and V9^CRIPT^ are found to make most of the stronger interactions with PDZ3 as labeled with the on mark along the Fig. 6(a) diagonal, consistent with their determining roles in substrate selectivity.^54,55^ The backbone sites of I327 on β_2_ turn on 
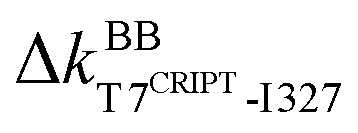
 and 
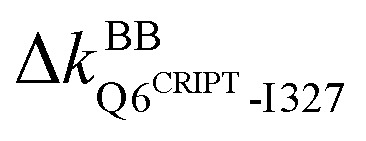
 to anchor the substrate, while its neighbor N326 mediates 
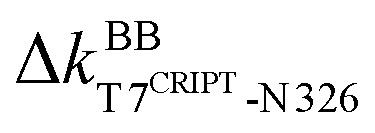
 and 
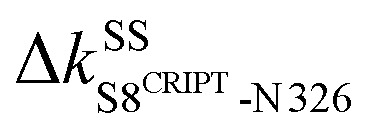
, Fig. 4(b) and 6. Stiffening responses (
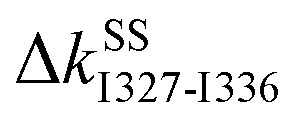
 and 
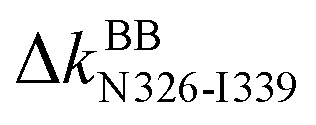
) then proceed on the other side of β_2_ facing β_3_. Next, the mechanical relay further passes to β_4_ (Δ*k*^BB^_I338-D357_ softening) and to the β_6_ end facing CT-extension α_3_ (Δ*k*^BB^_G356-K393_ stiffening). Following this route across the β-sandwich, the coupling of Y392 side chain at the C-terminal edge of β_6_ with G410 in between β_7_ and β_8_, Δ*k*^BS^_Y392-G410_, is off, leading to detachment of the β_7_–β_8_ hairpin. This route of mechanical relay seeding from the T7^CRIPT^-I357 coupling in the substrate binding groove and reaching CT-extension is colored green in Fig. 4(b) and 6. The inter-domain allostery of CRIPT binding can thus be understood as the chain of physically contiguous residues exhibiting significant coupling strength variation.

Another route starts on the β_2_ side of the binding groove. Turning on the prominent hydrophobic interaction 
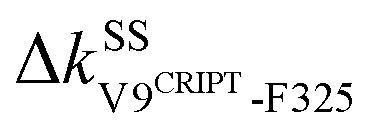
 in holoPDZ3 softens the β_2_–*l*_5_ coupling of Δ*k*^SS^_F325-L353 _in apoPDZ3 and a chain of softening responses (Δ*k*^BS^_E352-R312_ and Δ*k*^SS^_R312-D357_) colored purple in Fig. 4(b) and 6 follows. On the α_2_ side of the binding groove, on the other hand, turning on 
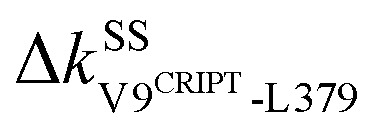
 leads to the mechanical relay route colored orange consisting mostly of stiffening responses that reaches the distal I388 in β_6_. Intra-domain communication in PDZ3 can thus proceed through multiple pathways. The mechanical coupling variation also provides comprehensive mechanistic basis for the NMR observed rigidification in structural flexibility after substrate binding.^6^

### 3.5 Mutation sensitive sites affecting the substrate binding of PDZ3 participate in mechanical relay

Although most of the prominent mechanical responses upon binding CRIPT are stiffening, certain inter-residue couplings soften with pronounced levels, indicating heterogeneity and complexity in the interaction network reorganization. By performing high-throughput mutagenesis over the β-sandwich residues, several sites not at the substrate-binding groove were shown to still have significant impact on CRIPT binding.^51^ In comparing the rigidity graphs of holoPDZ3 and apoPDZ3 all-atom MD simulations, most of the mechanically responsive residues away from the binding groove, *i.e.*, on the mechanical relay routes shown in Fig. 4(b) and 6, predict the mutation sensitive sites identified experimentally, including I336 and I338 in β_3_, L353 in *l*_5_, V362 in β_4_, and I388 in β_6_. Our results indicate that the experimentally measurable functional property of PDZ3 residues as their extents of imposing long-rang effects on substrate binding can be linked to the specific variation in their interaction strengths during the protein dynamics at different substrate binding states. The property of mechanical relay thus provides the mechanistic basis for propagation of substrate binding signals as altered coupling strengths. Certain residues such as I338 exhibit responses in both its backbone and side-chain couplings, showcasing the cooperation of chemical components. In another experimental work, R318 not captured by the high-throughput mutagenesis^51^ was found to affect CRIPT binding,^52^ and our rigidity graph analysis identifies it to also exhibit prominent mechanical response.

### 3.6 Mechanical relay of the inter-domain allostery in PDZ3 exhibit strong signals in multiple sequence alignment

For PDZ3, inter-domain allostery is functionally more relevant but much less understood. Our analysis of rigidity graphs at different substrate binding states shows that the two processes are actually linked and hence provides a unified view. The intra-domain routes of mechanical relay all reach the edges of β_1_ (R312), β_4_ (G356 and D357), and β_6_ (Y392 and K393) that face the CT-extension as revealed in their coupling strength responses, and this cluster links to the Δ*k*^BS^_Y392-G410 _knob that drives the inter-domain allostery for the detachment of the β_7_–β_8_ hairpin from the β-sandwich, Fig. 4(b) and 6.

Another prominent coupling of CT-extension with β-sandwich is between Y397 in α_3_ and S339 in β_3_, and the latter is on the green route of mechanical relay. Unlike the turning off of Δ*k*^BS^_Y392-G410 _upon substrate binding, Δ*k*^BS^_S339-Y397 _is neutral and the coupling persists with high strengths in both holoPDZ3 and apoPDZ3. Y397 phosphorylation has been shown to affect the inter-domain allostery with SH3,^30^ and our result points to the disruption of this coupling as a likely mechanism. Although α_3_ remains linked to β_3_ with Δ*k*^BS^_S339-Y397 _neutral, the β_7_–β_8_ detachment upon Δ*k*^BS^_Y392-G410 _turned off leads to significant reorganization in the mechanical couplings in α_3_, particularly at the F400 site facing the β-sandwich. For the residues conducting inter-domain allostery as predicted by the rigidity graph analysis, their functional relevance is further analyzed by designing a multiple sequence alignment (MSA) limited to α_3_-containing PDZ3 analogs as described in Materials and methods. D357, the mostly conserved residue in the β-sandwich-only MSA,^56^ is found to be preserved among the α_3_-bearing sequences of PDZ3, and the other key residues for the inter-domain allostery observed in all-atom MD simulations, R312, the Y392 knob, Y397, and F400 are all very highly conserved when CT-extension is considered in MSA, Fig. S2.† The mechanical relay identified here for PDZ3 inter-domain allostery thus exhibits strong signals in the MSA concerning the sequences that contain the CT-extension α_3_.

## 4 Conclusions

In both RT and PDZ3, comparing the rigidity graphs of protein dynamics at different substrate binding states unravels specific sets of spatially contiguous residues as routes of mechanical relay. In such a connection over distal sites, the inter-linked couplings include backbone as well as side chains with an intricate coordination of their polar and hydrophobic interactions. For residues exhibiting prominent responses in the reorganization of the protein interaction network, the close correspondence with the experimentally identified functional sites and the highly conserved spots in our simulation-motivated MSA indicates that the mechanical relay system is under significant evolutionary pressure. From the explicit-solvent all-atom MD simulations conducted at different molecular binding states, comparing their residue rigidity graphs hence provides a useful approach to understanding the protein functional properties in terms of specific molecular interactions. In many important cases, the mechanical coupling network of a substrate like DNA also exhibits complicated behaviors.^13^ Capturing the coupled reorganization over the rigidity graphs of the enzyme–substrate pair is likely key to understand the sequence-specific functional properties^57^ such as binding affinities, catalytic mechanism, and kinetics. In these complex scenarios, the framework of our structure-mechanics statistical learning and the data structure of graphical analysis are readily applicable.

## Data availability

The Python code for construction of rigidity graphs, identification of prominent modes, and quantification of mechanical responses due to substrate association/dissociation can be found at https://github.com/nixnmtm/MechanicalRelay.

## Author contributions

NR: conceptualization, methodology, software, writing – original draft preparation; TC: methodology and software; HY: conceptualization, writing – review & editing; JW: conceptualization, methodology, software, writing – original draft preparation, writing – review & editing, supervision.

## Conflicts of interest

There are no conflicts to declare.

## Supplementary Material

SC-013-D1SC06184D-s001

## References

[cit1] Changeux J. P. (2005). Science.

[cit2] Smock R. G., Gierasch L. M. (2009). Science.

[cit3] Latorraca N. R., Venkatakrishnan A. J., Dror R. O. (2016). Chem. Rev..

[cit4] Cooper A., Dryden D. T. (1984). Eur. Biophys. J..

[cit5] Petit C. M., Zhang J., Sapienza P. J., Fuentes E. J., Lee A. L. (2009). Proc. Natl. Acad. Sci. U. S. A..

[cit6] Lee A. L. (2015). Biophys. Rev..

[cit7] Cyphers S., Ruff E. F., Behr J. M., Chodera J. D., Levinson N. M. (2017). Nat. Chem. Biol..

[cit8] Meisburger S. P., Thomas W. C., Watkins M. B., Ando N. (2017). Chem. Rev..

[cit9] Banerjee-Ghosh K., Ghosh S., Mazal H., Riven I., Haran G., Naaman R. (2020). J. Am. Chem. Soc..

[cit10] Hanson J. A., Duderstadt K., Watkins L. P., Bhattacharyya S., Brokaw J., Chu J. W., Yang H. (2007). Proc. Natl. Acad. Sci. U. S. A..

[cit11] Brokaw J. B., Chu J.-W. (2010). Biophys. J..

[cit12] Morrell T. E., Rafalska-Metcalf I. U., Yang H., Chu J.-W. (2018). J. Am. Chem. Soc..

[cit13] Chen Y.-T., Yang H., Chu J.-W. (2020). Chem. Sci..

[cit14] Raj N., Click T., Yang H., Chu J.-W. (2021). Comput. Struct. Biotechnol. J..

[cit15] Bahar I., Lezon T. R., Bakan A., Shrivastava I. H. (2010). Chem. Rev..

[cit16] Zheng W. J., Brooks B. R., Thirumalai D. (2006). Proc. Natl. Acad. Sci. U. S. A..

[cit17] Thirumalai D., Hyeon C., Zhuravlev P. I., Lorimer G. H. (2019). Chem. Rev..

[cit18] Morcos F., Pagnani A., Lunt B., Bertolino A., Marks D. S., Sander C., Zecchina R., Onuchic J. N., Hwa T., Weigt M. (2011). Proc. Natl. Acad. Sci. U. S. A..

[cit19] Halabi N., Rivoire O., Leibler S., Ranganathan R. (2009). Cell.

[cit20] Rivoire O., Reynolds K. A., Ranganathan R. (2016). PLoS Comput. Biol..

[cit21] Tirion M. M. (1996). Phys. Rev. Lett..

[cit22] Haliloğlu T., Bahar I., Erman B. (1997). Phys. Rev. Lett..

[cit23] Atilgan A. R., Durell S. R., Jernigan R. L., Demirel M. C., Keskin O., Bahar I. (2001). Biophys. J..

[cit24] Stetz G., Verkhivker G. M. (2016). J. Chem. Inf. Model..

[cit25] Sprang S. R., Fletterick R. J., Gráf L., Rutter W. J., Craik C. S. (1988). Crit. Rev. Biotechnol..

[cit26] Evnin L. B., Vásquez J. R., Craik C. S. (1990). Proc. Natl. Acad. Sci. U. S. A..

[cit27] Vindigni A., Di Cera E. (1998). Protein Sci..

[cit28] Krem M. M., Prasad S., Di Cera E. (2002). J. Biol. Chem..

[cit29] Hedstrom L. (2002). Chem. Rev..

[cit30] Zhang J., Petit C. M., King D. S., Lee A. L. (2011). J. Biol. Chem..

[cit31] Pasternak A., Ringe D., Hedstrom L. (1999). Protein Sci..

[cit32] Doyle D. A., Lee A., Lewis J., Kim E., Sheng M., MacKinnon R. (1996). Cell.

[cit33] Best R. B., Zhu X., Shim J., Lopes P. E. M., Mittal J., Feig M., MacKerell J., Alexander D. (2012). J. Chem. Theory Comput..

[cit34] Abraham M. J., Murtola T., Schulz R., Páll S., Smith J. C., Hess B., Lindahl E. (2015). SoftwareX.

[cit35] Chu J.-W., Voth G. A. (2006). Biophys. J..

[cit36] Camara-Artigas A., Murciano-Calles J., Martínez J. C. (2019). Acta Crystallogr., Sect. D: Struct. Biol..

[cit37] Notredame C., Higgins D. G., Heringa J. (2000). J. Mol. Biol..

[cit38] Bódi A., Kaslik G., Venekei I., Gráf L. (2001). Eur. J. Biochem..

[cit39] Li X. F., Nie X., Tang J. G. (1998). Biochem. Biophys. Res. Commun..

[cit40] Várallyay E., Pál G., Patthy A., Szilágyi L., Gráf L. (1998). Biochem. Biophys. Res. Commun..

[cit41] Kromann-Hansen T., Lange E. L., Sørensen H. P., Hassanzadeh-Ghassabeh G., Huang M., Jensen J. K., Muyldermans S., Declerck P. J., Komives E. A., Andreasen P. A. (2017). Sci. Rep..

[cit42] Goettig P., Brandstetter H., Magdolen V. (2019). Biochimie.

[cit43] Pineda A. O., Chen Z.-W., Bah A., Garvey L. C., Mathews F. S., Di Cera E. (2006). J. Biol. Chem..

[cit44] Gandhi P. S., Chen Z., Mathews F. S., Di Cera E. (2008). Proc. Natl. Acad. Sci. U. S. A..

[cit45] Niu W., Chen Z., Gandhi P. S., Vogt A. D., Pozzi N., Pelc L. A., Zapata F., Di Cera E. (2011). Biochemistry.

[cit46] Sichler K., Kopetzki E., Huber R., Bode W., Hopfner K.-P., Brandstetter H. (2003). J. Biol. Chem..

[cit47] Eigenbrot C., Ganesan R., Kirchhofer D. (2010). FEBS J..

[cit48] Várallyay E., Lengyel Z., Gráf L., Szilágyi L. (1997). Biochem. Biophys. Res. Commun..

[cit49] Hedstrom L., Perona J. J., Rutter W. J. (1994). Biochemistry.

[cit50] Peterson F. C., Gordon N. C., Gettins P. G. (2001). Biochemistry.

[cit51] McLaughlin J., Richard N., Poelwijk F. J., Raman A., Gosal W. S., Ranganathan R. (2012). Nature.

[cit52] Chi C. N., Engström A., Gianni S., Larsson M., Jemth P. (2006). J. Biol. Chem..

[cit53] Zhang J., Lewis S. M., Kuhlman B., Lee A. L. (2013). Structure.

[cit54] Gianni S., Haq S. R., Montemiglio L. C., Jürgens M. C., Engström Å., Chi C. N., Brunori M., Jemth P. (2011). J. Biol. Chem..

[cit55] Luck K., Charbonnier S., Trave G. (2012). FEBS Lett..

[cit56] Lockless S. W., Ranganathan R. (1999). Science.

[cit57] Venkatramani R., Radhakrishnan R. (2008). Phys. Rev. Lett..

