# Supplementary material for: Structure-mechanics statistical learning uncovers mechanical relay in proteins
Source: Chem Sci. 2022 Jan 19;13(13):3688–96. doi: 10.1039/d1sc06184d (PMC8966636; doi:10.1039/d1sc06184d)
Supplement: SC-013-D1SC06184D-s001 [file SC-013-D1SC06184D-s001.pdf]

Electronic Supplementary Material (ESI) for Chemical Science.
This journal is © The Royal Society of Chemistry 2022

Supporting Information for Structure-Mechanics Statistical Learning Uncovers Mechanical Relay in Proteins

Nixon Raj,† Timothy H. Click,† Haw Yang,‡ and Jhih-Wei Chu*,¶

†*Institute of Bioinformatics and Systems Biology, National Yang Ming Chiao Tung University, Hsinchu 30010, Taiwan, ROC*

‡*Department of Chemistry, Princeton University, Princeton, NJ 08544, USA*

¶*Institute of Bioinformatics and Systems Biology; Department of Biological Science and Technology; Institute of Molecular Medicine and Bioengineering; Center for Intelligent Drug Systems and Smart Bio-devices (IDS^{2}B), National Yang Ming Chiao Tung University, Hsinchu 30010, Taiwan, ROC*

E-mail: jwchu@nctu.edu.tw

Supporting Information Available

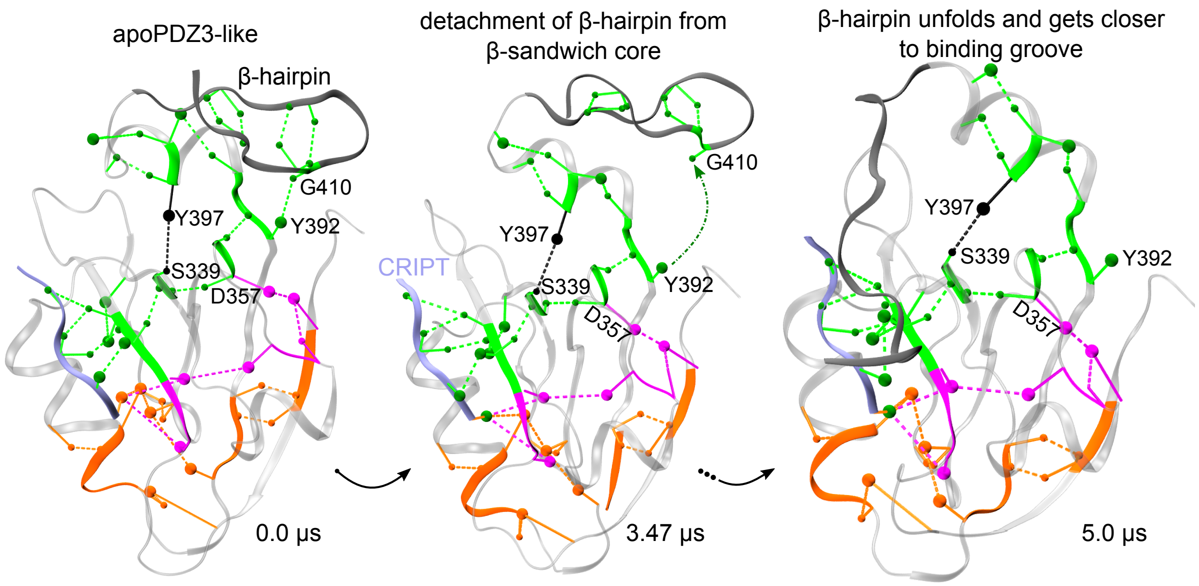

Figure S1: The inter-domain conformational change in PDZ3 upon CRIPT binding. Left: the initial structure of apoPDZ3. Center: the β-hairpin detachment ($\Delta k_{\text{G410-Y392}}$ off) from the β-sandwich core. Right: the unfolding of the β_7-β_8 hairpin that comes close to the peptide binding groove. The β_7-β_8 hairpin is colored in gray, while the other secondary structures displayed in transparent silver. The three mechanical relay systems are also shown following the coloring scheme in Fig. 4 and Fig. 6(b). The CRIPT peptide is colored in ice-blue. The important Y397-S339 interaction persisting throughout the conformational change is shown in black.

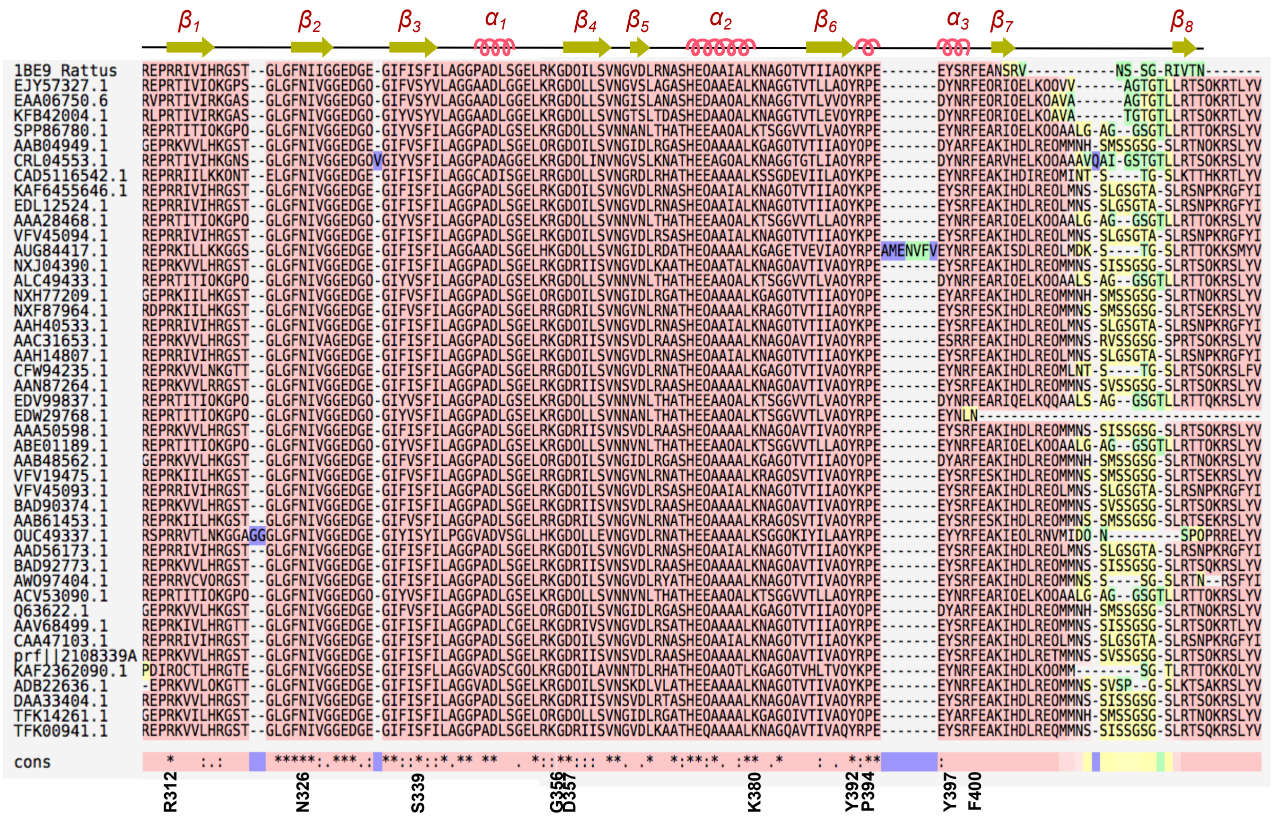

Figure S2: Multiple sequence alignment for the PDZ3 sequences containing the CT-extension $\alpha3$. The prominent rigidity modes that contain residues involved in the mechanical communication with $\alpha3$, i.e., inter-domain allostery, are highlighted to illustrate their very high levels of conservation. The asterisk label at bottom indicates the fully conserved residues in the MSA.
